# Bovine spongiform encephalopathy and variant Creutzfeldt-Jakob disease: background, evolution, and current concerns.

**DOI:** 10.3201/eid0701.010102

**Published:** 2001

**Authors:** P Brown, R G Will, R Bradley, D M Asher, L Detwiler

**Affiliations:** National Institute of Neurological Disorders and Stroke, National Institutes of Health, Bethesda, Maryland, USA. brownp@ninds.nih.gov

## Abstract

The epidemic of bovine spongiform encephalopathy (BSE) in the United Kingdom, which began in 1986 and has affected nearly 200,000 cattle, is waning to a conclusion, but leaves in its wake an outbreak of human Creutzfeldt-Jakob disease, most probably resulting from the consumption of beef products contaminated by central nervous system tissue. Although averaging only 10-15 cases a year since its first appearance in 1994, its future magnitude and geographic distribution (in countries that have imported infected British cattle or cattle products, or have endogenous BSE) cannot yet be predicted. The possibility that large numbers of apparently healthy persons might be incubating the disease raises concerns about iatrogenic transmissions through instrumentation (surgery and medical diagnostic procedures) and blood and organ donations. Government agencies in many countries continue to implement new measures to minimize this risk.


### Bovine Spongiform Encephalopathy and Variant Creutzfeldt-Jakob Disease: Background, Evolution, and Current Concerns

**Paul Brown,* Robert G. Will,† Raymond Bradley,‡ David M. Asher,§ and Linda Detwiler¶**
*National Institute of Neurological Disorders and Stroke, National Institutes of Health, Bethesda, Maryland, USA; †National Creutzfeldt-Jakob Disease Surveillance Unit, Western General Hospital, Edinburgh, Scotland; ‡Central Veterinary Laboratory, New Haw, Addlestone, UK; §Center for Biologics Evaluation and Research, Food and Drug Administration, Rockville, Maryland, USA; ¶Animal and Plant Health Inspection Service, U.S. Department of Agriculture, Robbinsville, New Jersey, USA

The epidemic of bovine spongiform encephalopathy (BSE) in the United Kingdom, which began in 1986 and has affected nearly 200,000 cattle, is waning to a conclusion, but leaves in its wake an outbreak of human Creutzfeldt-Jakob disease, most probably resulting from the consumption of beef products contaminated by central nervous system tissue. Although averaging only 10-15 cases a year since its first appearance in 1994, its future magnitude and geographic distribution (in countries that have imported infected British cattle or cattle products, or have endogenous BSE) cannot yet be predicted. The possibility that large numbers of apparently healthy persons might be incubating the disease raises concerns about iatrogenic transmissions through instrumentation (surgery and medical diagnostic procedures) and blood and organ donations. Government agencies in many countries continue to implement new measures to minimize this risk.

### Bovine Spongiform Encephalopathy

"The hungry Sheep look up, and are not fed,
But swoln with wind, and the rank mist they draw
Rot inwardly, and foul contagion spread…"
John Milton, *Lycidas* (1637)

Bovine spongiform encephalopathy (BSE) or "mad cow disease" appears to have originated from scrapie, an endemic spongiform encephalopathy of sheep and goats that has been recognized in Europe since the mid-18th century (1). It has since spread to most sheep-breeding countries and is widespread in the United Kingdom (UK), where until 1988 the rendered carcasses of livestock (including sheep) were fed to ruminants and other animals as a protein-rich nutritional supplement.

During rendering, carcasses from which all consumable parts had been removed were milled and then decomposed in large vats by boiling at atmospheric or higher pressures, producing an aqueous slurry of protein under a layer of fat (tallow). After the fat was removed, the slurry was desiccated into a meat and bone meal product that was packaged by the animal food industry and distributed to owners of livestock and other captive animals (e.g., zoo and laboratory animals, breeding species, pets).

Although elements of the ensuing story are still disputed (including its origin from scrapie, rather than from unrecognized endemic BSE), it appears likely that changes in the rendering process that had taken place around 1980 allowed the etiologic agent in infected carcasses to survive, contaminate the protein supplement, and infect

Address for correspondence: Paul Brown, Building 36, Room 4A-05, National Institutes of Health, 36 Convent Drive, MSC 4122 Bethesda, MD 20892-4122; fax: 301-496-8275; e-mail: brownp@ninds.nih.gov.

cattle. Cattle carcasses and carcass wastes were then recycled through the rendering plants, increasing the levels of the now cattle-adapted pathogen in the protein supplement and eventually causing a full-scale BSE epidemic (2-5).

Recognition of this source of infection has led to a series of countermeasures taken by the UK and other countries to break the cycle of cattle reinfection, restrict the geographic spread of disease, and eliminate potential sources of new infections (Figure, Appendix). Probably the single most important measure in the UK was the imposition in 1988 of a ruminant protein feed ban that by 1992 began to bring the epidemic under control. However, the loss of nearly 200,000 diseased cattle, followed by preemptive slaughter and destruction of nearly four and a half million asymptomatic cattle >30 months of age, has crippled the British livestock industry and also affected the tallow, gelatin, and pharmaceutical industries, all of which make bovine-derived products.

BSE is not restricted to the UK. Cases have occurred in many other countries as a result of imported live animals or livestock food supplements (Table 1). In some countries, including the UK, the incidence of new cases is decreasing, but in other countries–France, Portugal, Germany, Spain, and the Republic of Ireland–the incidence appears to be increasing, or initial cases have only recently appeared. The explanation for this phenomenon is most probably improved case ascertainment (supported by active surveillance and immunologic methods), but new infections from contaminated feed intended for other species (e.g., pigs and poultry) may also be a contributing factor. Although in many countries, BSE has been identified in native-born cattle, no indigenous index case has been reported outside the UK (i.e., no case originating de novo or from cow-to-cow transmission). Whatever the origin of these cases, recycling of their contaminated tissues through livestock feed supplements could have occurred in the same way as in the UK.

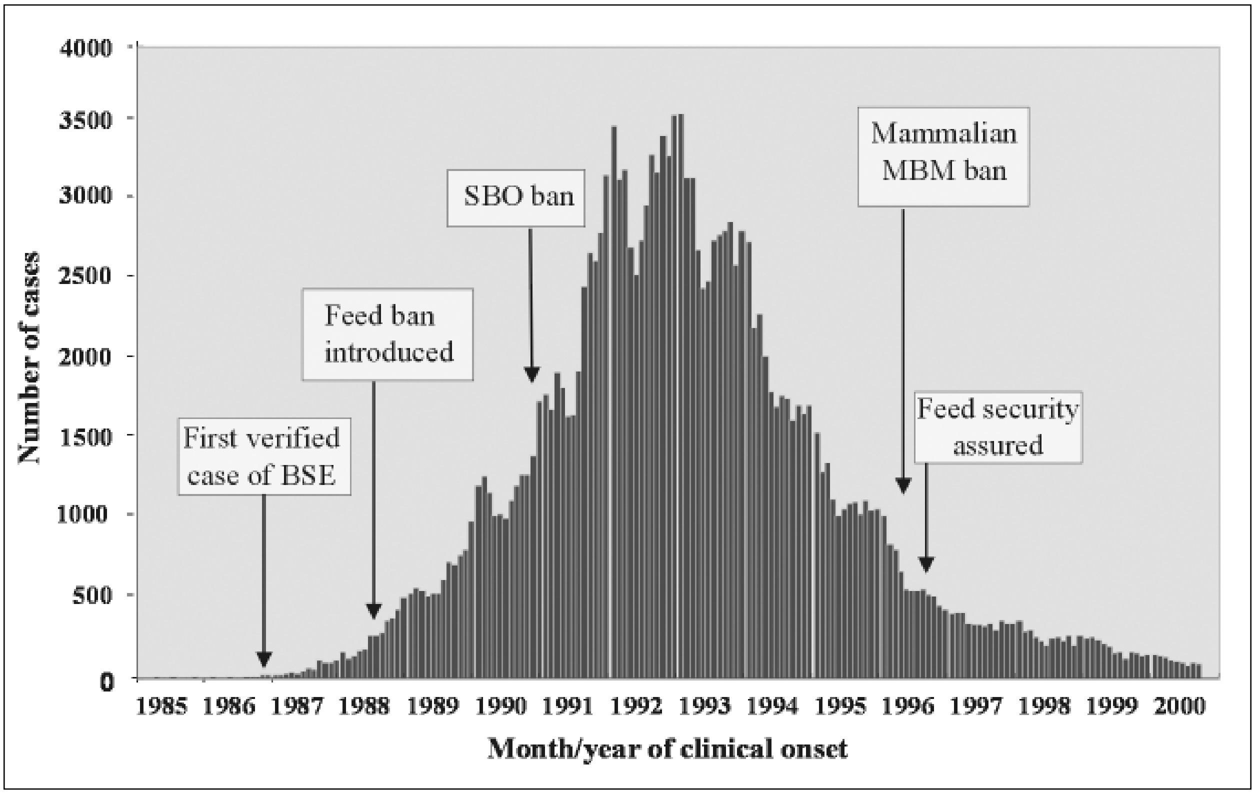

Figure. Time course of epidemic bovine spongiform encephalopathy in the United Kingdom, 1986-2000, with dates of major precautionary interventions. The mammalian ban on meat and bone meal in March 1996 extended a 1994 ban for farmed food animal species to include all mammalian species.
SBO = specified bovine offals (brain, spinal cord, thymus, tonsil, spleen, and intestines from cattle >6 months of age); MBM = meat and bone meal (protein residue produced by rendering).

Table 1. Reported cases of bovine spongiform encephalopathy in the United Kingdom and other countries (as of December 2000)

| Country | Native cases | Imported cases | Total cases |
|---|---|---|---|
| United Kingdom | 180,376[a] | - | 180,376 |
| Republic of Ireland | 487 | 12 | 499 |
| Portugal | 446 | 6 | 452 |
| Switzerland[b] | 363 | - | 363 |
| France[b] | 150 | 1 | 151 |
| Belgium | 18 | - | 18 |
| Netherlands | 6 | - | 6 |
| Liechtenstein | 2 | - | 2 |
| Denmark | 1 | 1 | 2 |
| Luxembourg | 1 | - | 1 |
| Germany | 3 | 6 | 9 |
| Oman | - | 2 | 2 |
| Italy | - | 2 | 2 |
| Spain[c] | - | 2 | 2 |
| Canada | - | 1 | 1 |
| Falklands (UK) | - | 1 | 1 |
| Azores (Portugal)[d] | - | 1 | 1 |

Data from Organization of International Epizootics (Paris) and Ministry of Agriculture, Fisheries, and Food (UK).
[a]Includes 1,287 cases in offshore British islands
[b]Includes cases detected by active surveillance with immunologic methods
[c]Origin and dates of imported cases are under investigation.
[d]Case imported from Germany.

BSE has not occurred in the United States or other countries that have historically imported little or no live cattle, beef products, or livestock nutritional supplements from the UK. Even though rendering procedures in other countries underwent changes similar to those in the UK during the late 1970s, BSE has apparently emerged solely within the UK. The most plausible explanation is that the proportion of sheep in the mix of rendered animal carcasses and the proportion of scrapie infections in such sheep were probably higher in the UK than elsewhere. These proportions were apparently sufficient to bring very low levels of the etiologic agent in batches of rendered carcasses over the threshold of transmission in the UK but not in other countries (5). An alternative explanation proposed in the recent Report of the BSE Inquiry (6) is that a pathogenic mutation occurred in cattle in the 1970s.

Either of these two hypotheses satisfies the need for an etiologic “seed” to survive the altered rendering process and escalate through recycling of an ever-larger number of infected carcasses. However, the bovine origin hypothesis assumes that a mutation occurred only in the UK and not in other countries where similar rendering processes would also have led to epidemic BSE if mutations were occurring. In humans, mutations have occurred all over the world, not just in the UK, and there is no reason to suppose that humans differ in this respect from other mammalian species. It would therefore be peculiar if the UK had the misfortune to host the cattle world’s only mutation.

### Variant Creutzfeldt-Jakob Disease (vCJD)

> How soon hath Time, the subtle thief of youth,
> Stol’n on his wing my three and twentieth year!
> John Milton, *Sonnet* (1632)

Within weeks of identification of the first case of BSE, concern was expressed about human risk (7-13), and as the epidemic unfolded, a series of measures was taken to eradicate BSE and prevent potentially infected tissues from reaching the human food chain (Appendix). A surveillance unit to monitor CJD was established in the UK in May 1990, and 3 years later, surveillance was extended to several other European countries, coordinated through the European Union. By this means it was hoped that any change in the epidemiology of CJD in the UK could be detected quickly and that the significance of the change could be assessed by comparison with the epidemiology of CJD in continental Europe.

Concern was heightened by the discovery that some exotic zoo ungulates, as well as domestic and captive wild cats, were becoming infected (14-18). The ungulates and domestic cats had also been fed diets supplemented by meat and bone meal, and the wild cats had been fed uncooked tissues, including cattle heads and spines. The possibility could therefore not be ignored that the disease might also cross the species barrier to humans from the consumption of beef or dairy products, or perhaps from occupational contact with cattle by ranchers, dairymen, or slaughterhouse workers.

What muted concerns about human infection was the presumption that BSE originated from scrapie, and scrapie was not a human pathogen. Nevertheless, even those who considered human risk to be remote acknowledged that scrapie might unpredictably show an altered host range after passage through cattle. Experimental precedents for such behavior were well known: passage of mouse-adapted strains of scrapie through hamsters altered their transmissibility on back passage to rodents (19,20); human strains of kuru or CJD did not transmit to ferrets or goats until passaged through primates or cats (21); and a bovine strain of BSE did not transmit to hamsters until passaged through mice (22). Alternatively, if BSE originated from a spontaneous mutation in cattle, experimental studies of species susceptibility to this new strain of transmissible spongiform encephalopathy (TSE) had not sufficiently advanced to predict that humans would not be susceptible. Nevertheless, during the 10 years after the first case of BSE was identified, cases of CJD did not increase in groups at high risk and continued to occur in the general population with the same spectrum of clinical and neuropathologic features as before the appearance of BSE.

Then, from May to October 1995, the CJD Surveillance Unit was notified of three cases of CJD in patients 16, 19, and 29 years of age (23,24). On neuropathologic examination, all three patients had amyloid plaques, which was unexpected in view of their occurrence in only 5%-10% of sporadic cases of CJD. The comparative youth of the patients and this unusual neuropathologic finding prompted a search for similar features in patients whose deaths might have been attributed to other diagnoses. In particular, cases of subacute sclerosing panencephalitis (SSPE) were scrutinized in view of a report from Poland that cases of CJD in three young patients had been identified by SSPE surveillance (25). No such cases were found in a review of the UK SSPE register.

If CJD in young patients was not being obscured by misdiagnosis, perhaps it reflected increased physician awareness through publicity surrounding BSE and iatrogenic CJD in recipients of contaminated growth hormone, or the active CJD surveillance program instituted in the UK, or the availability of genetic and proteinase-resistant protein (PrP) immunocytochemistry. Although all these factors may have contributed to ascertainment bias, most of the excess cases were in older age groups, in which CJD was now being diagnosed more often than in earlier decades.

By December 1995, the Surveillance Unit had been informed of 10 suspected cases of CJD in persons <50 years of age. Some were found to have sporadic or familial CJD or some other disease; however, two of the patients, ages 29 and 30 years, were later confirmed neuropathologically to have CJD and, like the previous three CJD patients, had extensive plaque deposition. As of January 1, 1996, the relationship between these cases and BSE began to excite suspicion but remained tentative because critical information judged necessary to establish a probable connection was still missing (Table 2).

During January, two additional cases of CJD in young patients were neuropathologically confirmed, and a distinctive clinical syndrome associated with plaque formation was beginning to emerge: young age at onset, early psychiatric symptoms, prominent ataxia, absence of periodic electroencephalographic activity, and a comparatively prolonged illness. However, each of these features, alone or in combination, may also be seen in classic sporadic or familial CJD. Caution was further justified by a review of the records of pre-1980 CJD patients in the UK, which identified three young patients who shared some of these features, and by the results of an inquiry about young patients with CJD in other European countries, which showed an age distribution similar to that in the UK. A major concern was that these seven apparently similar cases might represent a heterogeneous group of patients with sporadic and familial forms of CJD.

Table 2. Evolving assessment of criteria used to link bovine spongiform encephalopathy and variant Creutzfeldt-Jakob disease.

| | Assessment through early 1996 | | | | |
|---|---|---|---|---|---|
| Criteria | Jan 1 | Feb 1 | Mar 1 | Mar 8 | Mar 20 |
| Novel clinical phenotype | Uncertain | Possible | **Probable** | Probable | Probable |
| Novel neuropathologic phenotype | Uncertain | Possible | **Probable** | Probable | Probable |
| Distinct from pre-1980 cases in UK | Unknown | Possible | **Probable** | Probable | Probable |
| No association with PRNP mutations | Uncertain | Uncertain | Uncertain | **Probable** | Probable |
| Distinct from cases outside UK | Unknown | Unknown | Unknown | Possible | **Probable** |

Full comparative neuropathologic examination of both pre- and post-1980 cases of CJD in young persons was needed, along with *PRNP* gene sequence analysis of as many cases as possible.

During February 1996, an additional case was referred to the Surveillance Unit with a clinical evolution similar to that of the previous seven patients, and neuropathologic examination of recent and historical cases confirmed that the recent cases were indeed distinctive. In particular, a morphologically unusual form of plaque was present in all cases: the florid or "daisy" plaque in which an amyloid core was surrounded by "petals" of spongiform change. As of March 1, despite the likelihood that this group of patients had a "new variant" of CJD, it was still unclear whether mutations were involved and whether such a syndrome was also occurring outside the UK–both points essential to confirming the association of this variant disease with exposure to BSE.

On March 4, genetic analysis was completed for six of the cases, and no pathogenic mutation was identified. These results effectively ruled out a genetic cause for the syndrome (although they did not rule out a genetic predisposition) and left the only remaining uncertainty–the geographic distribution of the variant phenotype–to be resolved by the European CJD surveillance system. The answer came by March 20: none of the young CJD patients in other European countries had the clinical and neuropathologic features of the UK cases. In the preceding week, two more variant cases had been neuropathologically confirmed, and a report on the entire group of 10 cases concluded that an unrecognized variant of CJD occurring only in persons <45 years of age was probably due to exposure to BSE (26).

This link has now been convincingly established in laboratory studies showing identical, distinctive biological and molecular biological features of the pathologic agent isolated from BSE-infected cattle and human cases of vCJD (27-29). The source of contamination appears to have been beef. However, muscle has never been reproducibly shown to contain the infectious agent in any form of spongiform encephalopathy, whatever the affected species, and thus, infection most probably resulted from beef products contaminated by nervous system tissue. Contamination could have occurred in any of the following ways: cerebral vascular emboli from cranial stunning instruments used to immobilize cattle before killing by exsanguination; contact of muscle with brain or spinal cord tissue by saws or other tools used during slaughter; inclusion of paraspinal ganglia in cuts of meat containing vertebral tissue (e.g., T-bone steaks); and perhaps most importantly, the presence of residual spinal cord and paraspinal ganglia tissue in the paste of "mechanically recovered meat" (a carcass compression extract) that could legally be added to cooked meat products such as meat pies, beef sausages, and various canned meat preparations. Measures have since been taken to eliminate these sources of potential contamination and limit the consequences of any contamination that may already have occurred (Appendix).

Although the amount of infectious tissue ingested must be a critical determinant for the transmission of BSE to humans in the form of vCJD, the human genotype at polymorphic codon 129 of the PRNP gene appears to play an important role in susceptibility to infection. The encoding alternatives, methionine (Met) and valine (Val), are distributed in the general Caucasian population in the approximate proportions of 50% Met/Val, 40% Met/Met, and 10% Val/Val. All 76 vCJD patients tested have been homozygous for methionine, and the apparently single infecting strain of BSE may not be able to replicate in any other human genotype. However, it is also possible that (as in the analogous oral infection of kuru and in peripheral iatrogenic CJD infections) heterozygotes are comparatively resistant to disease and become ill after longer incubation periods than those of homozygotes (30-33).

### Predictions about the vCJD Outbreak

Think not but that I know these things; or think
I know them not: not therefore am I short
Of knowing what I ought.

John Milton, *Paradise Regained* (1671)

The onset of illness in the first case of vCJD occurred in early 1994, nearly a decade after the first case of BSE was recognized in cattle. Assuming that the earliest appearance of vCJD reflects the earliest exposure to BSE, this incubation period is consistent with those following peripheral infections in experimental animals and in cases of iatrogenic CJD in humans. Through the end of November 2000, the overall tally was 87 definite or probable cases of vCJD in the UK, 2 confirmed and 1 probable case in France, and a single confirmed case in the Republic of Ireland (Table 3). The Irish patient had lived for some years in England; however, none of the French patients had lived in or visited the UK, so their infection must have come either from beef or beef products imported from the UK (approximately 5%-10% of the beef consumed in France) or from BSE-affected cattle in France. From a European standpoint, it would be much more troubling if imported beef were the source, as most European countries also imported beef or beef products from the UK, although in smaller quantities.

Unlike the BSE epidemic, the vCJD outbreak has shown only a modest increase during its first 6 years, and the number of cases with onsets in 2000 remains well below the previous year's total, although additional cases will certainly be identified in coming months. The difference between BSE and vCJD may be due to the fact that, in humans, recycling of infected tissue has not occurred, and thus the epidemic will evolve much more slowly than in cattle, or the difference may indicate a limited outbreak in humans due to very small infectious doses that, except in genetically susceptible persons, cannot surmount the combined effects of a species barrier and comparatively inefficient route of infection.

Much of the lingering uncertainty about the extent of the vCJD outbreak is attributable to the fact that the incubation period of vCJD is unknown. If the average incubation period is 10 to 15 years, the earliest patients with vCJD would have been infected in the early 1980s, when BSE was still silently incubating in small but increasing numbers of cattle. In this case, the large increase in human exposure to contaminated tissues during the late 1980s could lead to a parallel increase in cases of vCJD during the next few years. If, however, the average incubation period of vCJD is 5 to 10 years, the earliest human infections would have begun in the mid- to late 1980s, when exposure to BSE was maximal. In this case, the outbreak of vCJD should remain small because of measures to eliminate both animal and human exposure to BSE instituted from 1987 to 1997. Depending on assumptions about the incubation period and other variables, mathematical modeling predicts that the total extent of the outbreak could range from fewer than one hundred to hundreds of thousands of cases (34-37).

If large numbers of infected persons are silently incubating the disease, the potential for human-to-human iatrogenic spread of vCJD is very real. Such apparently healthy persons would be subject to the same kinds of medical and surgical procedures experienced by the general population, including endoscopies, vascular catheterizations, operations for trauma or illness, and blood and organ donations. If, as suspected, the amount and distribution of the infectious agent in tissues of persons with vCJD is greater than in other forms of CJD, the exposure of medical and surgical instruments to possibly infectious internal tissues and the transfer of tissues as grafts and transplants become a matter of much greater concern than the nearly negligible risk currently posed by cases of sporadic CJD.

Table 3. Chronology of variant Creutzfeld-Jakob disease (vCJD) in the United Kingdom and other European countries, as of December 2000

| Year of onset | United Kingdom | France | Ireland |
|---|---|---|---|
| 1994 | 8 | 1 | |
| 1995 | 10 | | |
| 1996 | 11 | | |
| 1997 | 14 | | |
| 1998 | 17 | | |
| 1999[a] | 20 (+4) | 1 (+1) | 1 |
| 2000[a] | 1 (+2) | | |

[a]Parentheses indicate still-living persons with probable vCJD or deceased persons whose diagnoses have not yet been confirmed by neuropathologic examination. In 2000, additional cases have been identified that do not yet meet the minimum clinical criteria for a premortem diagnosis of "probable" vCJD. Dates are for year of onset of illness, not year of death.

### Recent and Future Policy Decisions

> A little onward lend thy guiding hand
> To these dark steps, a little further on…
>
> John Milton, *Samson Agonistes* (1671)

Several governments have implemented policies to minimize the risk for human-to-human disease transmission through blood donations from apparently healthy persons who may be in the incubation phase of vCJD. In the

UK, where whole blood or blood products from some persons who later died of vCJD have been administered to others, all plasma is imported and all blood from UK donors is filtered to eliminate leukocytes, which are the most likely carriers of infectivity in blood (38-40). In the United States, a blood donor policy excludes donations from anyone who has lived in or visited the UK for a cumulative period of 6 months or more during 1980 to 1996. The 6-month period was based on the fact that >80% of total US person-years in the UK would be excluded and that the 2%-3% deficit of blood donors resulting from the deferral could be absorbed by the blood banking industry without undue shortages. Several countries (Canada, Australia, New Zealand, Switzerland, Japan, and Germany) have since applied these criteria and formulated similar policies.

Because of the possibility of widespread infection in the UK, concern extends beyond blood and organ donors to the safe use of medical and surgical instruments, particularly those used in neurosurgery and ophthalmic surgery. In the absence of a screening test, a zero-risk policy is untenable because it would require termination of the national organ donor program. A compromise might be the temporary deferral of organ donors–or perhaps only corneal donors–younger than 30 or 40 years of age. However, this measure might so diminish (and panic) the donor population as to be inadvisable. Similar considerations apply to invasive medical and surgical procedures: sound medical practice cannot be suspended on the basis of a theoretical risk for vCJD, and it would be unethical to deny needed procedures to persons suspected of having CJD. Under the circumstances, disposable instruments should be used whenever possible, and a standard sterilization protocol for reusable instruments should be implemented that includes the most stringent possible disinfectants (e.g., the combined use of 1 N sodium hydroxide and autoclaving at 134°C, as recommended in the recent World Health Organization guidelines on infection control for CJD [41]). No effective sterilization procedure yet exists for instruments or instrument parts too delicate to withstand these harsh measures. Each such instrument must be disinfected to the maximum extent possible, for example by washing repeatedly with detergent/proteinase solutions and exposing the washed instruments to less harsh chemicals (e.g., 6 M urea or 4 M guanidinium thiocyanate) that have shown moderate to good disinfection of TSE tissue extracts (42-44).

An equally important issue is whether the bovine-adapted scrapie agent has recrossed the species barrier to sheep, carrying its newly acquired ability to infect humans. The only reliable method to distinguish strains of TSE is a time-consuming comparison of incubation periods and topographic features of brain lesions after injection into different strains of inbred mice (28). Glycotyping of PrP strains extracted from diseased brain tissue is much faster but has not been convincingly shown to discriminate reliably between BSE and scrapie. Moreover, neither method has been used to test a sheep-adapted strain of BSE (that is, after multiple passages through sheep), which might have lost the distinguishing characteristics found on primary passage from cow to sheep.

If BSE did back-cross to sheep fed the same contaminated meat and bone meal that infected cattle, the consequences for humans will remain limited to the same period of risk as BSE–roughly 1980 through 1996–unless sheep BSE, like sheep scrapie, can be horizontally or maternally transmitted. Without a test to discriminate between the two diseases, there would be no defense against the development of endemic BSE in sheep and the consequent risk for human infection from sheep as well as cows. Therefore, global elimination of animal TSEs must seriously be considered.

Such a goal is more practical than it was even a few years ago. National programs to eliminate scrapie have historically relied on selective slaughter of blood lines or in some cases entire flocks in which scrapie was identified, and all such attempts have failed. Molecular genetic tools are now available to guide scrapie-resistance breeding programs that until recently depended on field observation and classical genetics, and immunologic tools can detect preclinical scrapie infection in tonsils, third eyelids, and possibly blood (45-48). The environmental durability of TSE pathogens will make their eradication difficult (49,50); however, the global elimination of TSE in sheep and other animals is a goal worth the expense, effort, and patience that will be needed for its achievement.

Dr. Brown is Senior Research Scientist in the Laboratory of Central Nervous System Studies at the National Institutes of Health. His most recent research focuses on the problem of iatrogenic Creutzfeldt-Jakob disease and on the potential for disease transmission through the administration of blood or blood products. He serves as consultant to the European CJD surveillance program and as Chair of TSEAC, the transmissible spongiform encephalopathy advisory committee of the United States Food and Drug Administration.

### References

1. Brown P, Bradley R. 1755 and all that: a historical primer of transmissible spongiform encephalopathy. Br Med J 1998; 317:1688-92.
2. Wells GAH, Scott AC, Johnson CT, Gunning RF, Hancock RD, Jeffrey M, et al. A novel progressive spongiform encephalopathy in cattle. Vet Rec 1987;121:419-20.
3. Collee JG, Bradley R. BSE: a decade on-Part 1. Lancet 1997; 349:636-42.
4. Collee JG, Bradley R. BSE: a decade on-Part 2. Lancet 1997; 349:715-21.
5. Brown P. The risk of bovine spongiform encephalopathy ("mad cow disease") to human health. JAMA 1997;278:1008-11.
6. The BSE inquiry: report, evidence and supporting papers of the inquiry into the emergence and identification of Bovine Spongiform Encephalopathy (BSE) and variant Creutzfeldt-Jakob Disease (vCJD) and the action taken in response to it up to 20 March 1996. Lord Phillips of Worth Matravers, Chairman. London: The Stationery Office. October 26, 2000.
7. Holt TA, Phillips J. Bovine spongiform encephalopathy. Br Med J 1988;296:1581-2.
8. BSE and scrapie: agents for change [editorial]. Lancet 1988;ii:607-8.
9. Taylor DM. Bovine spongiform encephalopathy and human health. Vet Rec 1989;125:413-5.
10. Dealer SF, Lacey RW. Transmissible spongiform encephalopathies: the threat of BSE to man. Food Microbiol 1990;7:253-79.
11. Kimberlin RH. Bovine spongiform encephalopathy: taking stock of the issues. Nature 1990;345:763-4.
12. Will RG. Is there a potential risk of transmission of BSE to the human population and how may this be assessed? In: Bradley R, Savey M, Marchant B, editors. Sub-acute spongiform encephalopathies. Dordrecht: Kluwer Academic Publishers; 1991. p. 179-86.
13. Brown P. The clinical epidemiology of Creutzfeldt-Jakob disease in the context of bovine spongiform encephalopathy. In: Bradley R, Savey M, Marchant B, editors. Subacute spongiform encephalopathies. Dordrecht: Kluwer Academic Publishers; 1991. p. 195-202.
14. Jeffrey M, Wells GAH. Spongiform encephalopathy in a Nyala (*Tragelaphus angasi*). Vet Pathol 1988;25:398-9.
15. Fleetwood AJ, Furley CW. Spongiform enecphalopathy in an eland. Vet Rec 1990;126:408-9.
16. Wyatt JM, Pearson GR, Smerdon T, Gruffydd-Jones TJ, Wells GAH. Spongiform encephalopathy in a cat. Vet Rec 1990;126:513.
17. Kirkwood JK, Wells GAH, Wilesmith JW, Cunningham AA, Jackson SI. Spongiform encephalopathy in an Arabian oryx (*Oryx leucoryx*) and a greater kudu (*Tragelaphus strepsiceros*). Vet Rec 1990;127:418-20.
18. Willoughby K, Kelly DF, Lyon DG, Wells GAH. Spongiform encephalopathy in a captive puma (*Felis concolor*). Vet Rec 1992;131:431-4.
19. Kimberlin RH, Cole S, Walker CA. Temporary and permanent modifications to a single strain of mouse scrapie on transmission to rats and hamsters. J Gen Virol 1987;68: 1875-81.
20. Kimberlin RH, Walker CA, Fraser H. The genomic identity of different strains of mouse scrapie is expressed in hamsters and preserved on reisolation in mice. J Gen Virol 1989;70: 2017-25.
21. Gibbs CJ Jr, Gajdusek DC, Amyx H. Strain variation in the viruses of Creutzfeldt-Jakob disease and kuru. In: Prusiner SB, Hadlow WJ, editors. Slow transmissible diseases of the nervous system. Volume 2. New York: Academic Press; 1979. p. 87-110.
22. Foster JD, Hope J, McConnell I, Bruce M, Fraser H. Transmission of bovine spongiform encephalopathy to sheep, goats, and mice. Ann NY Acad Sci 1994;724:300-3.
23. Britton TC, Al-Sarraj S, Shaw C, Campbell T, Collinge J. Sporadic Creutzfeldt-Jakob disease in a 16-year-old in the UK. Lancet 1995;346:1155.
24. Bateman D, Hilton D, Love S, Zeidler M, Beck J, Collinge J. Sporadic Creutzfeldt-Jakob disease in a 18-year-old in the UK. Lancet 1995;346:1155-6.
25. Kulczycki J, Jedrzejowska H, Gajkowski K, Tarnowska-Dziduszko E, Lojkowska W. Creutzfeldt-Jakob disease in young people. Eur J Epidemiol 1991;5:501-4.
26. Will RG, Ironside, JW, Zeidler M, Cousens SN, Estibeiro K, Alperovitch A, et al. A new variant of Creutzfeldt-Jakob disease in the UK. Lancet 1996;347:921-5.
27. Collinge J, Sidle KC, Heads J, Ironside J, Hill AF. Molecular analysis of prion strain variation and the aetiology of 'new variant' CJD. Nature 1996;383:685-90.
28. Bruce ME, Will RG, Ironside JW, McConnell I, Drummond D, Suttie A, et al. Transmissions to mice indicate that 'new variant' CJD is caused by the BSE agent. Nature 1997;389:498-501.
29. Scott MR, Will R, Ironside J, Nguyen HB, Tremblay P, DeArmond SJ, et al. Compelling transgenetic evidence for transmission of bovine spongiform encephalopathy prions to humans. Proc Natl Acad Sci U S A 1999;96:15137-42.
30. Cervenáková L, Goldfarb LG, Garruto R, Lee H-E, Gajdusek DC, Brown P. Phenotype-genotype studies in kuru: implications for new variant Creutzfeldt-Jakob disease. Proc Nat Acad Sci U S A 1998;95:13239-41.
31. Lee H-S, Brown P, Cervenáková L, Garruto RM, Alpers MP, Gajdusek DC, et al. Evidence for susceptibility of the 129MM PRNP genotype in epidemic kuru. J Infect Dis 2000, in press.
32. d'Aignaux JH, Costagliola D, Maccario J, Billette de Villemeur T, Brandel J-P, Deslys JP, et al. Incubation period of Creutzfeldt-Jakob disease in human growth hormone recipients in France. Neurology 1999;53:1197-201.

33. Brown P, Preece M, Brandel J-P, Sato T, McShane L, Zerr I, et al. Iatrogenic Creutzfeldt-Jakob disease at the millennium. Neurology 2000;55:1075-81.
34. Cousens SN, Vynnycky E, Zeidler M, Will RG, Smith PG. Predicting the CJD epidemic in humans. Nature 1997;385:197-8.
35. Ghani AC, Ferguson NM, Donnelly CA Hagenaars TJ, Anderson RM. Epidemiological determinants of the pattern and magnitude of the vCJD epidemic in Great Britain. Proc R Soc London (Series B) 1998;265:2443-52.
36. Donnelly CA, Ferguson NM. Predictions and scenario analysis for vCJD. In: Statistical aspects of BSE and vCJD: models for an epidemic. Boca Raton (FL): CRC Press LLC 1999:163-94.
37. Ghani AC, Ferguson NM, Donnelly CA, Anderson RM. Predicted vCJD mortality in Great Britain. Nature 2000;406:583-4.
38. Brown P. Can Creutzfeldt-Jakob disease be transmitted by transfusion? Curr Opin Hematol 1995;76:472-7.
39. Brown P, Cervenáková L, McShane LM, Barber P, Rubenstein R, Drohan WN. Further studies of blood infectivity in an experimental model of tranmissible spongiform encephalopathy, with an explanation of why blood components do not transmit disease in humans. Transfusion 1999;39: 1169-78.
40. Brown P, Cervenáková L. Reply to a letter to the editor. Transfusion 2000;40:754-5.
41. WHO infection control guidelines for transmissible spongiform encephalopathies: report of a WHO Consultation. WHO/CDS/CSR/APH/2000.3. Geneva: March 23-26, 1999.
42. Kimberlin RH, Walker CA. Competition between strains of scrapie depends on the blocking agent being infectious. Intervirology 1985;23:74-81.
43. Manuelidis L. Decontamination of Creutzfeldt-Jakob disease and other transmissible agents. J Neurovirol 1997;3:62-5.
44. Pocchiari M, Peano S, Conz A, Eshkol A, Maillard, Brown P, et al. Combination ultrafiltration and 6 M urea treatment of human growth hormone effectively minimizes risk from potential Creutzfeldt-Jakob disease virus contamination. Horm Res 1991;35:161-6.
45. Roels S, Vanopdenbosch E, Langeveld JP, Schreuder BE. Immunohistochemical evaluation of tonsillar tissue for preclinical screening of scrapie based on surveillance in Belgium. Vet Rec 1999;145:524-5.
46. O'Rourke KI, Baszler TV, Besser TE, Miller JM, Cutlip RC, Wells GA, et al. Preclinical diagnosis of scrapie by immunohistochemistry of third eyelid lymphoid tissue. J Clin Microbiol 2000;38:3254-9.
47. Schmerr MJ, Jenny AL, Bulgin MS, Miller JM, Hamir AN, Cutlip RC, et al. Use of capillary electrophoresis and fluorescent labeled peptides to detect the abnormal prion protein in the blood of animals that are infected with a transmissible spongiform encephalopathy. J Chromatog A 1999;853:207-14.
48. Brown P, Cervenáková L, Diringer H. Blood infectivity and the prospects for a diagnostic screening test in Creutzfeldt-Jakob disease. J Lab Clin Med. In press 2001.
49. Palsson PA. Rida (scrapie) in Iceland and its epidemiology. In: Prusiner SB, Hadlow WJ, editors. Slow transmissible diseases of the nervous system. Volume 1. New York: Academic Press; 1979. p. 357-66.
50. Brown P, Gadjusek DC. Survival of scrapie virus after 3 years' interment. Lancet 1991;337:269-70.

**Appendix**

Table A. Measures taken to prevent the spread of bovine spongiform encephalopathy to animals

| Precautions | Great Britain[a] | European Union[a] | United States |
|---|---|---|---|
| BSE made a notifiable disease | June 1988 | Apr 1990 | Nov 1987 |
| BSE surveillance, with histologic examination of brains | June 1988 | May 1990 | May 1990 |
| Ban on ruminant protein in ruminant feed | July 1988 | | |
| Ban on export of UK cattle born before July 1988 feed ban | | July 1989 | |
| Ban on import of live ruminants and most ruminant products from all BSE countries | | | July/Nov 1989 |
| Ban on export of UK cattle >6 months of age | | Mar 1990 | |
| Ban on SBO for use in animal nutrition; ban on export of SBO and feed containing SBO to EU countries | Sept 1990 | | |
| High-risk waste to be rendered at 133°C/3 bar/20 min (or other approved procedure) | | Nov 1990 | |
| Ban on export of SBO and feed containing SBO to non-EU countries | July 1991 | | |
| Ban on MBM from SBO in fertilizer | Nov 1991 | | |
| After Jan 1, 1995, rendering methods must sterilize BSE | | June 1994 | |
| Ban on mammalian MBM in ruminant feed | | July 1994 | |
| BSE surveillance includes immunohistologic features of brains | | | Oct 1993 |
| Ban on mammalian protein in ruminant feed[b] | Nov 1994 | | Aug 1997 |
| Ban on import of live ruminants and most ruminant products (including meat products) from all countries of Europe | | | Dec 1997 |
| Immunologic testing for ruminant protein in animal feed | | July 1995 | |
| Mammalian MBM prohibited from all animal feed/fertilizer | Mar/Apr 1996 | | |
| Slaughtered cattle >30 months old (except certain beef cattle >42 months old) ruled unfit for animal use (hides for leather excluded) | Mar 1996 | | |
| Mammalian MBM and MBM-containing feed recalled | June 1996 | | |
| All mammalian waste to be rendered at 133°C/3 bar/20 min (or other approved procedure) | | July 1996 | |
| Cattle tracing system improved | Sept 1998 | | |
| Quarantine of 3 sheep flocks imported from Europe with possible exposure to BSE (4 animals die with atypical TSE) | | | Oct 1998 |
| BSE surveillance of fallen stock (downer cows) is intensified | | | Oct 1998 |
| Proposal to eradicate scrapie is rejuvenated | | | Nov 1999 |
| Allow export of deboned beef from cattle >30 months old born after July 1996 | Aug 1999 | | |
| Prohibit use of animal protein, including MBM and blood meal (but excluding milk, or fish meal for nonruminants) in feed for any farmed animal species (effective January 1, 2001) | | Dec 2000 | |
| Prohibit importation of rendered protein and rendering wastes originating or processed in Europe | | | Dec 2000 |

[a]In Northern Ireland and Scotland, dates of implementation sometimes differed from those shown for England and Wales; in addition, individual European Union countries often adopted different measures on different dates.
[b]Some exemptions, e.g., milk, blood, and gelatin.
BSE: bovine spongiform encephalopathy; EU = European Union; MBM = meat and bone meal (protein residue produced by rendering); SBO = specified bovine offals (brain, spinal cord, thymus, tonsil, spleen, and intestines from cattle >6 months of age); TSE = transmissible spongiform encephalopathy.

Table B. Measures taken to prevent the spread of bovine spongiform encephalopathy to humans

| Precautions | Great Britain[a] | European Union[a] | United States |
|---|---|---|---|
| Compulsory slaughter of BSE-affected cattle | Aug 1988 | | |
| Destroy milk from affected cattle (except for milk fed to cows' own calves) | Dec 1988 | | |
| Ban on import of UK cattle born after July 1988 feed ban | | July 1989 | |
| Ban on SBO for domestic consumption | Nov 1989 | | |
| Ban on export to EU of SBO and certain other tissues, including lymph nodes, pituitaries, and serum | Apr 1990 | Apr 1990 | |
| Ban on export of live UK cattle (except calves <6 months old) | June 1990 | June 1990 | |
| Ban on use of head meat after skull opened | Mar 1992 | | |
| FDA recommends use of BSE/scrapie-free sources for materials used in dietary supplements; request for safety plans | | | Nov 1992 |
| Cell lines used for biologicals should be BSE agent-free | | | May 1993 |
| FDA requests that bovine source materials (except gelatin) used in manufacture of regulated products be restricted to BSE-free countries | | | Dec 1993 |
| Bone-in beef only from farms with no BSE for 6 years; if not BSE-free, must be deboned with visible nervous and lymphatic tissue removed | | July 1994 | |
| FDA requests that bovine-derived materials for animal use or for cosmetics and dietary supplements not be sourced from BSE countries | | | Aug 1994 |
| Thymus and intestines from calves <6 months old made SBO | Nov 1994 | | |
| Import of beef only from UK cattle 1) >30 months, or 2) from herds BSE-free for 6 years, or 3) if not BSE-free, deboned with visible nervous tissue and specified lymph nodes removed | | July 1995 | |
| SBO ban broadened to include whole skull (SBM) | Aug 1995 | | |
| MRM from bovine vertebral column banned and export prohibited | Dec 1995 | | |
| Removal of lymph nodes and visible nervous tissue from bovine meat >30 months exported to EU | Jan 1996 | | |
| Ban on export of all UK cattle and cattle products except milk | | Mar 1996 | |
| SBM ban broadened to include entire head (excluding uncontaminated tongue) | Mar 1996 | | |
| Slaughtered cattle >30 months (or certain beef cattle >42 months) ruled unfit for animal or human use (hides excepted) | Mar 1996 | | |
| FDA urges manufacturers of FDA-regulated human products to take steps to assure freedom from BSE agent | | May 1996 | |
| Partial lifting of export ban on tallow and gelatin | | June 1996 | |
| SBM ban broadened to include certain sheep and goat heads, spleens, and spinal cords (SRM) | Sept 1996 | | |
| FDA recommends withdrawal of plasma and plasma products made from pools to which persons who later died of CJD had contributed | | | Dec 1996 |
| CNS tissues excluded from cosmetic products for use in EU | | Jan 1997 | |
| BSE cohort cattle in UK ordered slaughtered and destroyed | Jan 1997 | | |
| Proposed ban on SRM in cosmetics for use in EU (effective October 2000) | | July 1997 | |
| SBM controls for cosmetics and medicinal products | Mar 1997 | | |
| FDA request to manufacturers that no bovine gelatin from BSE countries be used in injectable, implantable, or ophthalmic products; and that special precautions be applied to gelatin for oral and topical use | | | Sept/Dec 1997 |
| Ban on marketing cosmetic products containing SRM prepared before April 1, 1998 | | Mar 1998 | |
| Allow export of beef and beef products from cattle >30 months in certified BSE-free herds from Northern Ireland | | Mar 1998 | |
| Importation of all plasma and plasma products for use in UK | Aug 1998 | | |
| FDA limits plasma product withdrawals to pools at risk for contamination by vCJD donors | | | Sept 1998 |
| Slaughter and destruction of offspring born to BSE-affected cattle after July 1996 | Jan 1999 | | |
| FDA guidance to defer blood donors with >6 months cumulative residence in UK during 1980-1996 | | | Nov 1999 |
| Leukodepletion of whole blood donations from UK residents | Jul/Nov 1999 | | |
| Public FDA discussion about possible risk associated with vaccines produced with bovine-derived materials from BSE countries | | | July 2000 |
| Withdrawal and destruction of a potentially tainted 1989 lot of polio vaccine from one manufacturer | Oct 2000 | | |
| SRM ban implemented (effective October 2000) | | July 2000 | |
| Ban on slaughter techniques that could contaminate cattle carcasses with brain emboli (e.g., pithing or pneumatic stun guns), effective Jan 2001 | | July 2000 | |
| All cattle >30 months old must have brain examinations for proteinase-resistant protein (PrP) before entering the food chain (effective Jan-Jun 2001) | | Dec 2000 | |

[a]In Northern Ireland and Scotland, dates of implementation sometimes differed from those shown for England and Wales; in addition, individual EU countries often adopted different measures on different dates.

CNS = central nervous system; EU = European Union; MRM = mechanically recovered meat; SBM = specified bovine materials (SBO plus entire head, including eyes but excluding tongue); SBO = Specified bovine offals (brain, spinal cord, thymus, tonsils, spleen, and intestines from cattle >6 months old); SRM = specified risk materials (SBM plus sheep and goat heads and spleens from animals of any age, and spinal cords from animals >1 year old).
